# Supplementary material for: Chromothripsis during telomere crisis is independent of NHEJ, and consistent with a replicative origin
Source: Genome Res. 2019 May;29(5):737–49. doi: 10.1101/gr.240705.118 (PMC6499312; doi:10.1101/gr.240705.118)
Supplement: Supplemental Material [file supp_gr.240705.118_Supplemental_file_1.zip › contigs/annotated_contigs/DB48/contig.2.DB48_length_927_mean_cov_6.67961165049.docx]

**DB48_length_927_mean_cov_6.67961165049**

```
ATCCACAATACCAACAACAATAACCCAAATCCTCCCAATTAAGAGGTGAAGTGGCGAGAGACATACTGAGGAACCAAATAAATTTACAA
 >chr7:52789744-52790180 + E=3e-226
GCTAAATGTGCTTAATGTTGATAAAACAAACCAAAGAAAGATGGTTTTACTTTGTATTTTTTGCCTTTCTAGTTGTGTTTGTTTGATTT

TACTTTTTATTTATGAATATGCATTCCTTTGAGATTTAAGAGAAACAATATTTTTGAAAAGACAGAATACTTAAAAAGTAAATACTTTT

CCAATAAGGGCTTGTTCTCTGCTTTATAAATGATGTCTCTGTCTGCACCCTCAATGAGTGAGTAATCAAGCTCCACCAGGCCTGTTTCA

CAGGGGCACTGGTCCCATTCATTCAGGCTCTGCCCTCATGACCTTACCACCTCCTAAAGGCCCTACCTCTTAATGCCG|TT|TGGCAGT
                                                                               >chr6_db
ACTGAGGACATAGCTTCACTAAAGAAGCTCTTTCAGACTCTTTCATGCTTCTTGGAGTCCTAGACTTCAAGATGAACAACTCAGGCCAA
b_hap3:3684845-3685340 - E=4e-249
GAGCTTGGGATGGCTGGCATTGTATCTCATTTAGAGAAAATGAGAGGAGAAACTCTGAGGTTCAACACTCTCAACACTGGAGAAGGATG

AAAGGCTGTATCTTGAAACACAGGTAGTAGTTAGTGGAAATAGTGGGCTGTAAAGGAGCTACCTTCTTCTCATCTCTACCATCTTATAT

ATGACAAGAGATTGGAGTTGACTAATGGACACACCTCATTTGAGGATTCAGGTCAATGAATATGAGAGAGTATAATGGGCAAAGCCTAT

TTTTTTTTTTTAGACAGAGTCTTGCTCTGTCGCCCAAGCTGGAGTTGGTGGCGTGATCTCAGCTCATTGTAATCTCCGCCCCCCGGGTT

CAAGCGATTCTCCTGCCTCAGCCTCCTGAGTAGCTGGGA
```
